# Colonic malakoplakia in a pediatric renal transplant recipient case report

**DOI:** 10.1177/2050313X241239866

**Published:** 2024-03-18

**Authors:** Kristen G Valencia Deray, Richard Kellermayer, Alexis C Gomez, Kalyani R Patel, Peace Imani, Seiji Kitagawa, Claire E Bocchini, Alvaro Orjuela

**Affiliations:** 1 Division of Infectious Diseases, Department of Pediatrics, Baylor College of Medicine, Houston, TX, USA; 2 Division of Pediatric Gastroenterology, Hepatology and Nutrition, Department of Pediatrics, Baylor College of Medicine, Houston, TX, USA; 3 Division of Pediatric Nephrology, Department of Pediatrics, Boston Children’s Hospital, Boston, MA, USA; 4 Department of Pathology & Immunology, Baylor College of Medicine, Houston, TX, USA; 5 Division of Pediatric Nephrology, Department of Pediatrics, Baylor College of Medicine, Houston, TX, USA

**Keywords:** Malakoplakia, renal transplant recipient, pediatric, recurrent gastrointestinal infections

## Abstract

Malakoplakia is a rare, chronic granulomatous disease that mainly affects the genitourinary system of immunocompromised adults. It is caused by a bactericidal deficit in macrophages and, therefore, the treatment includes antimicrobials that reach high concentrations in macrophages. To our knowledge, we present the first case of malakoplakia in a pediatric solid organ transplant recipient. Our patient is a 15-year-old male renal transplant recipient who presented with recurrent diarrhea. Blood, urine, and gastrointestinal pathogen panel testing were positive for enteroaggregative *Escherichia coli.* A colonoscopy revealed diffuse malakoplakia. He had a complete resolution of symptoms with trimethoprim-sulfamethoxazole therapy. Unfortunately, his malakoplakia recurred after 9 months prompting the transition of therapy to oral gentamicin with subsequent remission. Malakoplakia should be considered in the differential of solid organ transplant recipients with recurrent gastrointestinal infections.

## Introduction

Malakoplakia is a chronic granulomatous disease that most commonly affects the genitourinary tract where it clinically presents as recurrent urinary tract infections (UTI).^1^ It is caused by a bactericidal deficit in macrophages and, therefore, the treatment includes antimicrobials that reach high concentrations in macrophages.^2^ Although it is described in immunocompetent hosts, immunosuppression is a well-known risk factor with up to 40% of cases being in patients with immunocompromise.^2^ We present a case of malakoplakia presenting as chronic diarrhea in a renal transplant recipient.

## Clinical case

A 15-year-old male deceased donor renal transplant recipient was admitted with 2 months of abdominal pain, diarrhea, and an 8 kg weight loss. His history included end-stage renal disease secondary to focal segmental glomerulosclerosis (FSGS) necessitating transplant 1.5 years prior to admission (PTA). He was at intermediate risk (Donor+/Recipient+) for cytomegalovirus and Epstein–Barr virus. His induction therapy was antithymocyte globulin and methylprednisolone and his maintenance immunosuppression included tacrolimus (goal trough 4–6 ng/mL) and 500 mg (376 mg/m^2^/dose) of mycophenolate twice daily. His transplant course was complicated by delayed graft function and recurrence of FSGS requiring high-dose steroids, plasmapheresis, and rituximab (last dose 2 months PTA); he continued to receive pulse dosing methylprednisolone every 2 weeks and 20 mg of prednisone daily at the time of admission. Diarrhea at presentation was 6–12 episodes of watery, bloody stools per day. Abdominal pain was 7–9/10 and central and left-sided; the pain did not radiate and was worse with eating and at night.

**Figure 1.**
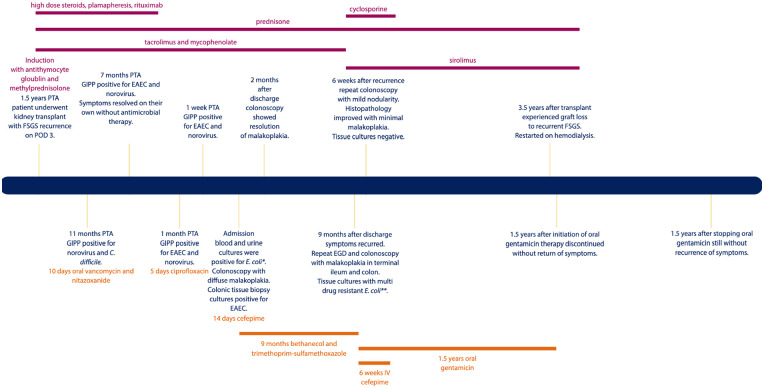
Timeline of clinical course post-transplant. The text in orange at the top of the figure includes immunosuppression medications. The text in green at the bottom of the figure denotes antimicrobial therapies. *C. difficile: Clostridioides difficile*; EAEC: enteroaggregative *Escherichia coli; E. coli: Escherichia coli*; EGD: esophagogastroduodenoscopy; FSGS: focal segmental glomerulonephritis; GIPP: gastrointestinal pathogen panel; POD: postoperative day; PTA: prior to admission. *E. coli isolates susceptible to cefepime, meropenem, amikacin, gentamicin, tobramycin, and trimethoprim/sulfamethoxazole and resistant to ceftriaxone, ciprofloxacin, and levofloxacin. **E. coli isolates susceptible to cefepime, meropenem, amikacin, gentamicin, tobramycin, and resistant to ceftriaxone, ciprofloxacin, levofloxacin, and trimethoprim/sulfamethoxazole.

**Figure 2.**
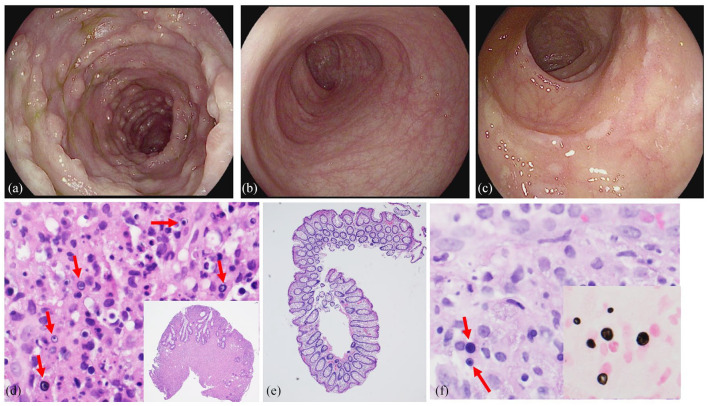
Colonoscopy and histopathologic findings of malakoplakia. (a) Diffuse blanching nodularity of the colon. (b) Grossly normal colonic mucosa. (c) White macular lesions in the colonic mucosa. (d) Panel D: Inset shows a lower power view of the colonic biopsy with mucosal expansion with sheets of histiocytes (HE, 40×). Panel D shows multiple concentrically layered or targetoid, basophilic inclusions, 2–10 µm in diameter thought to represent remnants of phagolysosomes mineralized by iron and calcium, as indicated by red arrows (H&E, 600×). Panel E: Normal colonic biopsy with complete resolution of malakoplakia (H&E, 40×). Panel F: Mucosal biopsy showing similar basophilic inclusions as indicated by red arrows (H&E, 600×). Panel F inset: These inclusions are referred to as Michaelis–Gutman bodies and are highlighted by a calcium stain, von Kossa (400×).

He had recurrent diarrhea for 11 months PTA for which he had four stool samples sent for infectious evaluation. The stool was persistently positive for norovirus and enteroaggregative *Escherichia coli* (*E. coli*; EAEC) starting at 11- and 7-month PTA, respectively (Figure 1). On admission, the patient was ill-appearing with tachycardia (heart rate 116 bpm). He had mild abdominal distension and diffuse tenderness without rebound or guarding. His hemoglobin was low at 9.5 g/dL and his white blood cell count was normal at 7 uL with 82% (32%–67%) neutrophils. Electrolytes, liver enzymes, and lipase were within normal limits. Creatinine was baseline at 0.99 mg/dL. Cytomegalovirus, enterovirus, Epstein- Barr virus, and herpes simplex virus were not detected by polymerase chain reaction (PCR) in blood. The gastrointestinal pathogen panel (Biofire FilmArray™ Gastrointestinal Panel by BioFire Diagnostics, Salt Lake City, UT, USA) had been obtained 1-week PTA and was therefore not repeated. Urine and blood cultures were positive for *E. coli* (blood isolate was PCR positive for EAEC). An abdominal computed tomography (CT) was obtained which showed very little intraluminal content in the intestines and fluid in the right colon. He underwent a colonoscopy on hospital day 2 which showed diffuse sessile polyposis from the sigmoid colon to the rectum (Figure 2). Histopathology from colonic biopsies revealed diffuse malakoplakia as evidenced by lamina propria expansion due to the proliferation of histiocytes containing laminated mineralized concretions called Michaelis–Gutman bodies (Figure 2). Biopsies from the small intestine, including the terminal ileum, did not show malakoplakia. Colonic tissue cultures were positive for EAEC. Notably, a viral culture was not performed on the colonic tissue to rule out possible involvement of norovirus.

He received 14 days of cefepime for bacteremia and urinary tract infection and bethanecol and trimethoprim–sulfamethoxazole twice daily for malakoplakia therapy which he continued at discharge. No changes were made to his immunosuppression regimen. He was discharged home on day 17 with a resolution of symptoms. Follow-up colonoscopy 2 months after discharge showed resolution of malakoplakia. Of note, aside from the CT scan, no further assessment was done of his genitourinary tract at this time as he did not have any urinary symptoms.

Symptoms recurred 9 months after initial discharge requiring re-admission. Repeat esophagogastroduodenoscopy and colonoscopy showed gross evidence of malakoplakia in the terminal ileum and colon. Histopathology confirmed recurrence involving the terminal ileum to the rectosigmoid colon despite adherence to therapies. Notably, the small intestine remained negative for malakoplakia. Tissue cultures grew multidrug-resistant *E. coli*, which was now resistant to trimethoprim–sulfamethoxazole. He was transitioned from trimethoprim–sulfamethoxazole, which he had been on for 9 months, to intravenous cefepime and oral gentamicin (based on susceptibilities). After 6 weeks of therapy, a repeat colonoscopy showed mild nodularity. Histopathology revealed near resolution in the ascending, descending, and rectosigmoid colon with minimal malakoplakia. However, there remained diffuse malakoplakia in the transverse colon. Tissue cultures were negative. He was discharged on oral gentamicin after a 2.5-month admission.

Two months after discharge, repeat esophagogastroduodenoscopy and colonoscopy were grossly normal. Histopathology confirmed the resolution of malakoplakia. After 1.5 years of oral gentamicin, therapy was discontinued without the return of symptoms for 1.5 years. His antibiotic duration was ultimately decided by his degree of immunosuppression. Unfortunately, our patient lost his graft to recurrent FSGS 3.5 years post-transplant and is now receiving hemodialysis. Once his graft was lost and his immunosuppression was decreased, we felt comfortable stopping his malakoplakia suppression therapy.

## Discussion

Malakoplakia is a rare chronic granulomatous disease that can affect most organ systems.^3^ It is uncommon in childhood with a mean age of 50 years at presentation, though cases range from 6 weeks to 88 years of age.^4^ Malakoplakia is increasingly described in adult solid organ transplant recipients (SOTR), with renal recipients being at the highest risk.^1^ To our knowledge, this is the first reported case of malakoplakia in a pediatric SOTR.

Malakoplakia primarily affects the genitourinary system and presents as a recurrent UTI. The gastrointestinal tract is the second most commonly affected system initially described in 1958.^5^ The descending and sigmoid colon and rectum are most commonly involved^5^; upper gastrointestinal tract disease has also been described.^6,7^ It can be diffuse or segmental and presents endoscopically in the following three ways: (1) unifocal mucosal tan to yellow nodules or plaques (most common), (2) multinodular or polypoidal lesions (our patient’s presentation), and (3) large mass lesions.^5^ Prior case reports indicate that CT findings of gastrointestinal malakoplakia are nonspecific, similar to our patient.^8,9^ Therefore, CT imaging may not be beneficial in the diagnosis.

Gastrointestinal malakoplakia can present with a spectrum of symptoms including diarrhea, abdominal pain, obstruction, or hemorrhage.^10^ Non-SOTR pediatric patients in the literature presented with bloody diarrhea, abdominal pain, rectal bleeding, loss of appetite, and weight loss, similar to our patient.^11^ The largest case series of gastrointestinal malakoplakia included 26 adult cases of which 64% were diagnosed incidentally on routine screening, indicating that patients can be asymptomatic.^7^

Diagnosis of malakoplakia is made by histopathologic findings of tissue infiltration by large, granular macrophages called von Hansemann cells^3^ which contain pathognomonic intracytoplasmic, calcified inclusion bodies called Michaelis–Gutmann bodies.^12^ These cells may require special stains such as von Kossa.^12,13^

Patients with malakoplakia are often immunosuppressed secondary to transplant, human immunodeficiency syndrome, malignancy, or autoimmune disease suggesting that immunosuppression plays a role in the pathogenesis.^1,3,6,9,13^ The main mechanism is a deficit of cyclic guanosine monophosphate (cGMP) in mononuclear cells. cGMP is necessary for lysosome function and its deficit leads to impaired bactericidal activity with incomplete removal of bacteria and persistence of bacteria in phagolysosomes.^14^ Schreiber and Maderazo found impaired monocyte activity against *E. coli* and *Staphylococcus aureus* in a patient with malakoplakia of the urinary tract and retroperitoneum.^15^ *E. coli* is the most commonly reported bacteria and was isolated in our patient. Other reported pathogens include *Klebsiella pneumoniae, Mycobacterium tuberculosis, Proteus* species, *Pseudomonas aeruginosa, Rhodococcus equi*, and *Staphylococcus aureus*.^5,12^

The therapeutic approach includes antimicrobials, such as trimethoprim–sulfamethoxazole and fluoroquinolones, that can reach high concentrations within macrophages where the bacteria are trapped in phagolysosomes.^5^ In addition, there has been success using the cholinergic agonist, bethanechol, which is thought to correct the lysosomal defect.^14^ The optimal duration of therapy has not been elucidated, though some reports suggest to use prolonged courses of antibiotics for improved graft outcomes and prevention of relapse.^2^ For patients who do not respond to antimicrobial therapy, surgical resection has been reported.^1^ Our patient had a long-term cure with an antibiotic (oral gentamicin) and bethanechol and, therefore, surgery was not considered. Biggar et al. showed that decreasing immunosuppression can also lead to clinical cure in some cases.^16^ We suggest following the resolution of symptoms as an indicator of clinical improvement and cure for gastrointestinal malakoplakia. As our patient’s disease relapsed, we also recommend having a low threshold to repeat colonoscopy with biopsy for worsening/relapsed symptoms.

## Conclusion

Malakoplakia is a rare granulomatous condition increasingly found in SOTR, especially renal transplant recipients. The most common presentation is recurrent UTI but gastrointestinal disease is also possible. Diagnosis is confirmed by histopathology. Gastrointestinal infections are quite frequent in children, including SOTR. However, a history of chronic or recurrent diarrhea particularly if with concurrent invasive *E. coli* bacteremia may need to prompt additional evaluation, including early endoscopy to rule out malakoplakia and avoid delay in management. Therapy includes decreasing immunosuppression, long-term antimicrobial therapy, and bethanechol.
